# The Choice of a Medical School

**Published:** 1908-09-05

**Authors:** 


### Notes on Medical Schools and Colleges.

### THE CHOICE OF A MEDICAL SCHOOL.

It is probable that by the time this number of The Hospital is published the great majority of those who are on the point of becoming medical students will already have made their choice of medical school, or that those who are responsible for their education will have done so for them.

Nevertheless, there are certainly some among the parents and guardians of prospective medical students who, even at the eleventh hour, have failed to reach a decision upon this somewhat important point. To the medical man who is sending his son into his own profession the choice is seldom a matter of difficulty or anxiety; but to parents, whose only means of information is the advice of the family doctor or the prospectuses of various rival medical schools, the whole subject is frequently beset with doubts and perplexities, and there is some excuse for them if they procrastinate until a decision is literally forced upon them by the arrival of the last week of September. It is for their benefit especially that these few remarks are offered.

We may suppose that the question, in the case of English students, of the desirability or the possibility of spending the first three years or so of the curriculum at one of the older Universities has already been settled. If it has not been settled we might say here that the opinion of very many of the best teachers and practitioners is strongly in favour of a preliminary course at Oxford or at Cambridge. The expenses are greater and the time occupied in mastering the early subjects is larger, but against these facts are to be reckoned many advantages, tangible as well as intangible. It is unnecessary to remind Scotsmen that the standard of medical education obtainable in the Scottish Universities is high and progressive, or to point out the debt which medicine, surgery, and midwifery owe, for example, to Edinburgh. The Irish schools also have long recognised the importance of developing and perfecting the methods of medical teaching, and with the forthcoming completion of the new Irish universities scheme there is every prospect of an even greater impetus being given to the facilities for medical education in Ireland. Beyond pointing out that the sister kingdoms possess the fullest equipment for scientific and clinical study, it is not necessary or desirable that we should offer further advice to those living in Scotland or Ireland upon the choice of a medical school. In each case the field is sufficiently wide, but the decision between the various Universities and schools of medicine is less difficult than it is in England. Of the provincial Universities of England we may say that in all of them there is evidence of an active and progressive spirit in the faculty of medicine, and that for many reasons their claims as medical schools should weigh heavily with those who live in their neighbourhood or who intend practising within the cities to which they are attached.

We now come to medical education in London. Here we would at once impress upon prospective students and their parents the vast importance of an early decision upon the question of obtaining a medical degree or being content with a diploma. At no time in the history of medicine has this question been more prominent than it is at the present moment. As things stand now, the medical student who starts out with the intention of obtaining diplomas to practise, and who subsequently finds that the possession of a degree will be all-important to him in his career, is in a most unfortunate position. We would advise every intending student to weigh carefully at the very outset of his curriculum the possibility and the advantages of entering for a University course. Even if, at a later date, circumstances arising from within or from without should compel him to give up the idea of a degree, he is free to change horses in mid-stream without incurring the traditional penalty for so doing. In other words, it is possible to take up the course of studies for the Conjoint Board Diplomas at the point at which the University of London course is relinquished; while the converse is impossible.

Having decided between the claims of Diploma and Degree, the next question is the claims of the individual medical schools of London. This consideration is scarcely less important to the University man who has completed his three years at Cambridge and has just arrived in London than to the boy fresh from school who is at the very beginning of his medical career. Nevertheless, although this decision is undoubtedly of considerable moment, it is desirable that too great significance should not be attached to it. Each hospital and medical school has its own individuality and its own traditions, and is an independent corporation; but, just as all the teaching hospitals are constituent schools of the University of London, so it may be said that all are good, and that within each may be obtained an excellent practical training in the art and science of medicine. No two medical schools are alike in detail, yet all are working along similar lines towards a similar object. This object is the turning out of efficient practitioners through the gateways of the examining bodies.

The usual broad classification of medical schools into large and small has several points in its favour, and at least affords a text for a few general considerations. Thus the larger schools, from their very size, are often more fully equipped with teachers and teaching material; their facilities for students' athletics and recreations are for the most part superior; and the student's choice of companions is wider. Moreover, it is said, and not without some reason, that in after life there are advantages in being a member of a large foundation whose influence and prestige have penetrated into every corner of the world. In favour of the smaller schools may be brought forward the many opportunities which exist therein for that personal contact between teacher and student which is so valuable in every form of education. However well organised and equipped the large medical school may be, it is difficult to believe that it can pay the same individual attention to the special requirements of each student as is paid in the smaller school. The smaller the institution the less is the chance of one of its members being lost sight of or neglected. Another point, which may or may not be an advantage, but which carries weight with the less ambitious, is the relative facility with which resident house appointments may be obtained in smaller hospitals by students after qualification. As for the contention that in the small medical schools there is more *esprit de corps* and good-fellowship among the students, while we can neither endorse it nor refute it, we believe that no such distinction between large and small schools can legitimately be drawn. Individual differences there certainly are between different hospitals in this respect, just as there are differences in respect of behaviour and "tone"; but they do not afford any grounds for contrasting the two groups of schools from this standpoint.

A factor which sometimes has considerable influence in the choice of a medical school is that curious temporary affinity which exists between certain public schools and certain hospitals and between certain colleges and certain hospitals. Boys and young men—no less than the rest of humanity—are liable to behave like sheep, and to save themselves the fatigue of independent judgment by following the track of their contemporaries. Fortunately in this instance there is little danger; but apart from other considerations, it is, perhaps, wiser for a man to enter a different hospital from the one which is being flooded with his school or college friends.

On the whole, it may be stated with confidence that at the present time, when competition between the various schools is desperately keen, an efficient education may be obtained by the industrious student of ordinary intelligence at any one of the London medical schools. Few men in after life regret their choice of a school, and he who fails at one is generally he who would fail at another. But, weighing one advantage against another and looking at the question from an impartial point of view, we would perhaps advise a young man of good ability and reasonable ambition, who is in doubt as to the best course to follow, to consider first the claims of the larger school.

